# Supplementary material for: Maternal Decisions on Portion Size and Portion Control Strategies for Snacks in Preschool Children
Source: Nutrients. 2019 Dec 9;11(12):3009. doi: 10.3390/nu11123009 (PMC6950145; doi:10.3390/nu11123009)
Supplement: Supplementary file 1 [file nutrients-11-03009-s001.pdf]

Table S1. Interview topic guide.

**Topic Guide**
1. *Can you tell me a little bit about how you normally decide how much food to serve your child at snack time?*
2. *And how did you decide today?*
3. *Is this amount similar or different to what you would normally provide?*
4. *Is there any reason why you might serve a different amount than you did today?*
5. *I can see you served more X than Y, can you explain your reasons for this?*
6. *Can you tell me about how these portion sizes are similar or different to other snack food items that you usually serve?*
7. *Can you tell me a little bit about how this amount is similar or different to the amount another family member would provide?*
8. *What role does your child play in deciding the amount of a snack that they receive?*
9. *If a snack comes in a packet for one, such as pom bears, how do you decide how much to give?*
10. *How does your child's liking/dislike of a snack influence the amount you serve?*
11. *How much of this snack would you expect your child to consume?*
12. *How do you decide how much you want your child to consume?*
13. *How would you normally respond if your child asks for more?*
14. *How would you normally respond if your child refuses a snack?*
15. *What information do you use when deciding on a portion size for yourself?*
16. *What information do you use to guide your decision regarding the amount of food to serve your child?*
17. *Can you tell me about a time you were provided with portion size information?*
18. *What is your opinion on receiving portion size guidance?*
19. *How willing would you be to follow portion size guidelines?*
20. *What is your opinion on supermarkets providing packaged snacks in a size suitable for child consumption?*
